# Factors Associated with SARS-CoV-2 Infection Evaluated by Antibody Response in a Sample of Workers from the Emilia-Romagna Region, Northern Italy

**DOI:** 10.3390/antib12040077

**Published:** 2023-12-01

**Authors:** Stefania Paduano, Michele Granata, Sara Turchi, Alberto Modenese, Pasquale Galante, Alessandro Poggi, Isabella Marchesi, Giuseppina Frezza, Giulia Dervishaj, Roberto Vivoli, Sara Verri, Simona Marchetti, Fabriziomaria Gobba, Annalisa Bargellini

**Affiliations:** 1 Department of Biomedical, Metabolic and Neural Sciences, Section of Public Health, University of Modena and Reggio Emilia, 41125 Modena, Italy; michele.granata@unimore.it (M.G.); sara.turchi@unimore.it (S.T.); alberto.modenese@unimore.it (A.M.); pasquale.galante1991@libero.it (P.G.); alle.poggi.90@gmail.com (A.P.); isabella.marchesi@unimore.it (I.M.); giuseppina.frezza@unimore.it (G.F.); dervishajgiulia@gmail.com (G.D.); fabriziomaria.gobba@unimore.it (F.G.); annalisa.bargellini@unimore.it (A.B.); 2 Test Laboratory, 41100 Modena, Italy; vivoli.roberto@laboratoriotest.it (R.V.); verri.sara@laboratoriotest.it (S.V.); marchetti.simona@laboratoriotest.it (S.M.)

**Keywords:** COVID-19, immune response, SARS-CoV-2, workers, antibody, case–control study

## Abstract

Factors associated with SARS-CoV-2 infection risk are still debated. This case–control study aims to investigate the possible relationship between SARS-CoV-2 infection, evaluated through antibody response, and the main sociodemographic, occupational, clinical-anamnestic, and biochemical factors in a population of Modena province (Northern Italy), mainly workers. Both workers who voluntarily joined the screening campaign proposed by companies and self-referred individuals who underwent serological testing were enrolled. Subjects with antibody positivity were recruited as cases (*n* = 166) and subjects tested negative (*n* = 239) as controls. A questionnaire on sociodemographic, occupational, and clinical data was administered through telephone interviews. Serum zinc/iron/copper/chromium/nickel, vitamins D/B12, folates, triglycerides, and LDL/HDL/total cholesterol were measured. Cases lived more often in urban areas (61.8% vs. 57%). Cases and controls did not differ significantly by working macrocategories, but the percentage of workers in the ceramic sector was higher among cases. Low adherence to preventive measures in the workplace was more frequent among seropositives. Folate concentration was significantly lower among cases. Therefore, adequate folate levels, living in rural areas, and good adherence to preventive strategies seem protective against infection. Workers in the ceramic sector seem to be at greater risk; specific factors involved are not defined, but preventive interventions are needed.

## 1. Introduction

The pandemic of Coronavirus Disease 2019 (COVID-19), caused by the severe acute respiratory syndrome coronavirus 2 (SARS-CoV-2) virus, has already caused more than 761 million infections and 6 million deaths globally [1]. Italy was the first European country severely affected by the pandemic, especially in the north of the country, with its first case of COVID-19 reported in February 2020 [2]. As of March 2023, more than 25 million cases of COVID-19 have been confirmed in Italy [3].

Many countries, including Italy, have implemented non-pharmaceutical policies such as lockdowns, social distancing, the use of face masks, personal hygiene measures, quarantine, and proper promotion of such strategies [2]. These strategies or non-observance of the mentioned measures can change the prevalence of the disease even months after the beginning of the pandemic [4].

In spring 2020, the WHO called for regional and national serosurveys, or the surveillance of confirmed cases, to estimate the extent of SARS-CoV-2 infection in the general population [5]. Several serosurveys have been conducted worldwide to assess the proportion of the population with antibodies against SARS-CoV-2 [6,7].

Real-time reverse transcriptase-polymerase chain reaction (real-time RT-PCR) is the gold standard molecular test for diagnosing SARS-CoV-2 infection. However, SARS-CoV-2 can induce innate and acquired immunity, resulting in the widespread inflammatory response to the disease [8] and the production of neutralizing antibodies against SARS-CoV-2 spike (S) glycoprotein or nucleocapsid (N) protein [9]. The seroprevalence of anti-SARS-CoV-2 antibodies may allow estimation of the total number of infections, identification of undetected asymptomatic individuals, and identification of population groups with an increased risk of infection [4,8,10].

Many studies have found higher infection risk among healthcare workers, who are known to be at greater risk of occupational exposure to biological agents, particularly infectious pathogens [11,12,13]. In general, the risk is considered higher for workers involved in providing services to the general public and for workers involved in activities with close physical proximity, especially in indoor settings or with shared transport or accommodation. Examples of such workers include the aforementioned healthcare workers, chemists, bank and supermarket employees, taxi drivers, and workers in the sports industry [14]. Other studies conducted in the province of Modena, Italy, reported similar results [2,10].

The role of habits, nutrients, trace elements, and clinical conditions in COVID-19 has been researched. Smoking has a controversial role in the development and severity of the infection [12,15,16,17]. COVID-19 patients with preexisting conditions such as diabetes, cardiovascular diseases, respiratory diseases, a history of cancer, dislipidemia, or obesity are at higher risk of severe disease [17,18,19,20]. Respiratory diseases have different impacts on the infection, with non-severe asthma suggested as being protective [16,21] and other diseases causing higher risk [22,23]. A higher risk is associated with dysregulation in the immune response, leading to a cytokine storm [24].

The potential preventive and therapeutic role of D and B vitamins has been investigated, as a possible immunomodulatory, anti-inflammatory, and antiviral action has been suggested [25,26,27]. The possible role of metals has also been of interest. Zinc and copper may have protective immunomodulatory and antiviral functions [28,29]. Iron is essential for high-load viruses, including SARS-CoV-2, which is inhibited by iron chelators in vitro [30]. Other trace elements, such as chromium and nickel, have been investigated as expressions of environmental and/or industrial pollution [31,32].

Currently, factors associated with the SARS-CoV-2 infection are still debated. The aim of this study is to assess the possible relationship between SARS-CoV-2 infection, evaluated through antibody response, and socio-demographic, occupational, clinical-anamnestic, and biochemical factors in a population recruited in Modena province, Northern Italy.

## 2. Materials and Methods

### 2.1. Study Population

In the early phases of the COVID-19 pandemic in Italy, due to the inadequate availability of rhino-pharyngeal swab testing for molecular diagnosis, a screening campaign for SARS-CoV-2 infection evaluated through antibody response was promoted both in the general population and in workers. A monocentric case–control study was conducted. Both workers who have voluntarily joined the screening campaign proposed by companies and self-referred individuals who underwent serological testing were enrolled at the Test Laboratory, one of the first private laboratories in Modena province to be authorized to carry out serological testing (Decree PG/2020/0307727 of 22 April 2020).

From October 2020 until May 2021, individuals aged > 18 years who were not vaccinated at the time of testing were included. Subjects with antibody positivity for IgM and/or IgG (cases) and a similar number of individuals negative to the same tests (controls) were selected, paired by sex, age, and Italian region or foreign country of birth. All enrolled persons provided informed consent for participation in the study and for processing their health data. This study was approved by the ethics committee “Area Vasta Emilia Nord” on 11 September 2020 (approval n. AUO/0024690/20).

### 2.2. Data Collection

A questionnaire survey was conducted among recruited subjects by telephone interview. An ad hoc questionnaire was designed with the aim of collecting information on the subject’s socio-demographic characteristics, including smoking habits and alcohol consumption, investigating the clinical anamnestic aspects, the employment sector, and the working conditions in the months preceding the serological test. The questionnaire was divided into three parts, each collecting different information:

1. Information on the subject’s socio-demographic characteristics, including smoking habits and alcohol consumption;
2. Clinical and medical aspects relevant to the study, including information on a possible diagnosis of SARS-CoV-2 infection and the presence of suspected symptoms for COVID-19 in the year preceding recruitment;
3. Information on the employment sector and the working conditions in the months prior to the blood draw, including the adoption of preventive measures to reduce SARS-CoV-2 transmission.

### 2.3. Laboratory Analysis

The anti-SARS-CoV-2 antibody titre in serum samples was determined by drawing 5 mL of venous blood for quantitative testing. The Elecsys^®^ kit (Roche Diagnostics, Risch-Rotkreuz, Switzerland) was used for the determination of anti-SARS-CoV-2 IgM and IgG by electro-chemiluminescence (ECLIA), with 100% sensitivity at 14 days after the onset of symptoms and 99.8% specificity. At the time of blood sampling, an aliquot of serum was frozen at −20 °C and subsequently used to analyze biochemical parameters, such as vitamin D (immunochemiluminescence method—kit 09038078190, Total Vitamin D III, Cobas Roche E601, Roche Diagnostics GmbH, Mannheim, Germany), vitamin B12 (immunochemiluminescence method—kit 07212771190 Cobas Roche E601, Roche Diagnostics GmbH, Mannheim, Germany), folates (immunochemiluminescence method—kit 07559992190 Cobas Roche E601, Roche Diagnostics GmbH, Mannheim, Germany), triglycerides, total cholesterol, HDL cholesterol (colorimetric method—kit 00018255740 ILAB Taurus, Instrumentation Laboratory Corporate Headquarters, Barcelona, Spain), LDL cholesterol (calculation), sideraemia (colorimetric method—kit 00018255740 ILAB Taurus, Instrumentation Laboratory Corporate Headquarters, Barcelona, Spain), zinc, copper, chromium, and nickel (method ICP-MS, Nexion 2000, PerkinElmer Inc., Waltham, MA, USA).

### 2.4. Statistical Analyses

Statistical analyses were carried out on all subjects in the study and stratified by subgroups of interest, such as gender, age group, and working macrocategory. Four age groups (<40 years, 40–49, 50–59, and ≥60), four living places (urban areas, rural areas, industrial areas, and others, such as seaside, mountain, and small urban/village areas), and twelve professional macrocategories divided according to the ISIC classification [33] were identified. The predominantly sedentary and office activities (ISIC J, K, L, M, and N sections) were grouped into one category, and the least represented (≤6 subjects) groups (ISIC A, D, F, H, and I sections) were merged into the “Other” category. The categories based on body mass index (BMI) were underweight (<18.5 kg/m^2^), normal weight (18.5–24.9 kg/m^2^), overweight (25.0–29.9 kg/m^2^), and obese (≥30 kg/m^2^) as defined by Forbes [34]. Cough, cold, sore throat, lack of strength, widespread pain in muscles and/or joints, low-grade or high fever, and inability/difficulty to taste and/or smell were considered the variable suspected symptoms for COVID-19, and if reported, other symptoms related to the disease were evaluated. The use of FFP2 masks and hand hygiene frequency were the preventive measures investigated in this study. A score of 0 was attributed to those who did not use a FFP2 mask or did so rarely, and to those who practiced hand hygiene less than 5 times a day. A score of 1 was attributed to those who wore a FFP2 mask regularly and practiced hand hygiene 5 or more times a day. The scores were then summed together to identify three different categories: 0 (low adherence: no or scarce adherence to the preventive strategies), 1 (moderate adherence: adoption of only one of the two investigated strategies), and 2 (high adherence: both frequent use of the FFP2 mask and hand hygiene ≥ 5 times a day). The data were summarized using descriptive statistics: absolute frequencies and percentages for qualitative variables; mean and standard deviation for normally distributed quantitative variables; and median and interquartile range for non-normally distributed quantitative variables. The differences between cases and controls were assessed using the χ2 test for categorical variables, the *t*-test for normally distributed continuous variables, and the Mann–Whitney test for those not normally distributed. The data were analyzed using the STATA software vs 15.1 (StataCorp, College Station, TX, USA). A two-sided *p*-value < 0.05 was considered statistically significant.

## 3. Results

From 1 October 2020 until 15 May 2021, 405 subjects were recruited (age 50.6 ± 11.9 years, range 19–71), mainly workers who underwent serological testing for anti-SARS-CoV-2 antibody testing at the Test Laboratory in Modena.

### 3.1. Characteristics of the Study Participants

Table 1 shows the main characteristics of the participants: 166 cases (age 50.6 ± 12.4 years, 42.8% male) and 239 controls (age 50.6 ± 11.6 years, 44.3% male).

Most of the population was aged between 40 and 59 years. Considering the BMI categories, there were no significant differences between cases and controls, but a slightly higher percentage of underweight and obese subjects was observed among cases. Subjects who smoked at least one cigarette a day were significantly lower in cases than in controls (11.5% vs. 20.2%). Most of the participants (*n* = 237) lived in urban areas, followed by those living in rural areas (*n* = 105) and in other areas (*n* = 53), such as seaside or mountain areas or small villages. Only 7 individuals reported living in industrial zones. Cases lived more often in an urban area (61.8% vs. 57%) or in an industrial area (3.7% vs. 0.4%) compared to controls (*p* = 0.04). Cases with a diagnosis of SARS-CoV-2 infection confirmed by swab were 70.3% vs. 12.7% of controls (*p* < 0.001). Among cases, 84.3% reported at least one suspected symptom for COVID-19 in the year before the blood draw, compared to 50.2% of controls (*p* < 0.001). The most frequent comorbidities were cardiovascular diseases. Arterial hypertension, valvulopathies, and arrhythmias were the most commonly reported cardiovascular pathologies. Among respiratory diseases, asthma, allergic rhinitis, and sinusitis were the most frequently reported. Previous cancers included melanoma, breast cancer, uterine cancer, thyroid cancer, urological cancer, and colon cancer. No significant differences among cases and controls were found.

The data regarding occupational categories showed no significant differences between cases and controls employed in the same work sector (Table 2). The most represented occupational macrocategory was “Manufacturing activities” (ISIC category C), in which “Ceramic”, “Chemical–pharmaceutical”, and “Metal–mechanical” were the most represented sectors (Table 3). The “Other” category (16.8%) included less represented sectors (such as the manufacture of food products, textiles, or wearing apparel). Considering the specific sectors, a higher percentage of cases were observed among ceramic workers. In contrast, a lower percentage, compared to controls, was observed in the chemical–pharmaceutical and metal–mechanical sectors.

Table 4 reports the participants’ adoption of preventive measures to reduce SARS-CoV-2 spread in their workplace. No or rare use of the FFP2 mask and hand hygiene less than 5 times a day were slightly more common behaviors among cases compared to controls, although no significant difference was recorded. Out of the participants who provided information on preventive measures, 167 (49.9%) showed moderate adherence to the investigated measures. Considering only the extreme categories (0 and 2), low adherence was more frequent among cases than controls (*p* = 0.06).

### 3.2. Biochemical Analyses

A total of 394 sera were analyzed (160 cases and 234 controls). The biochemical analyses of 11 participants were not carried out due to the remaining serum scarcity. The concentration of folate was found to be significantly lower in the cases’ sera. No significant differences emerged for the other parameters (Table 5).

## 4. Discussion

This case–control study investigated possible associations between socio-demographic, occupational, clinical-anamnestic, and biochemical factors and SARS-CoV-2 infection evaluated by antibody response in a population of the province of Modena and surrounding areas. Our data suggest a possible association between smoking habit, living place, folates, and SARS-CoV-2 infection evaluated through antibody response.

The percentage of smokers was significantly higher among controls, suggesting that smokers seem to be less susceptible to SARS-CoV-2 infection. These results are in line with other studies [12,35]. The reduced risk of SARS-CoV-2 infection among smokers has been hypothesized to be linked to the effect of nicotine on the nicotinic acetylcholine receptor (nAChR). This effect would result in a reduction in potential sites for viral entry into the pulmonary alveolar epithelium and the inhibition of pro-inflammatory cytokines [36,37]. However, the role of cigarette smoking in the SARS-CoV-2 infection is still debated. In a study conducted among sailors, smoking was associated with a lower susceptibility to SARS-CoV-2 infection. Nevertheless, 71% of smokers developed COVID-19, so it seems unreasonable to affirm that smoking strongly protects against COVID-19 in that population [38]. On the other hand, Eapen et al. suggest that smoking would facilitate the entry of the virus into the cell with a mechanism based on a significant increase in the expression of lysosomal proteases, i.e., lysosomal associated membrane protein 1 (LAMP-1) and catepsin-L, which facilitate the fusion of spike proteins with membranes under acidic pH conditions [15]. In addition, smoking is a strong risk factor for cardiovascular disease and chronic obstructive lung disease, which have been shown to be linked with increased severity and COVID-19-associated mortality [39,40]. Any conclusion regarding the possible protective role of smoking vs. SARS-CoV-2 infection should be treated with extreme caution by both the general population and healthcare professionals.

The living place may be associated with the SARS-CoV-2 infection, as evaluated by antibody responses. Individuals who live in urban or industrial areas are more likely to become infected, in line with other research [41,42]. This result suggests a relationship between pollution, population density, and the likelihood of infection, as reported by other authors [43,44].

Among cases, 70.3% were diagnosed with SARS-CoV-2 infection confirmed by swab, compared to 12.7% of controls. This finding can be traced back to a previous SARS-CoV-2 infection and the subsequent decrease in IgM and IgG levels over time, below the detection limit of the method [7]. To date, the issue of immunity duration in subjects positive for SARS-CoV-2 has been widely discussed. According to the literature, antibodies against SARS-CoV-2 were developed in over 90% of patients who were infected for the first time. However, several studies have found that antibody responses tend to decrease in the first few months after infection. The rate of decrease is very variable until non-detectable titres occur in 12–60% of subjects over a period of 8 weeks to 11 months. This decrease seems to depend on the severity of the disease and individual factors [45,46,47,48].

Among seropositive subjects, 84.3% reported at least one suspected symptom of COVID-19. The asymptomatic rate from our study (15.7%) fits in the range of 9.2–84.7%, as reported in other studies [42,49]. Asymptomatic cases seem to have a key role in driving the infection spread since virus transmission from asymptomatic subjects accounts for more than 50% of transmissions [50]. From our data, about 50% of controls declared having experienced symptoms compatible with COVID-19, which are often aspecific. In line with our findings, Struyf et al. conclude that neither the presence nor the absence of signs and symptoms can be used to confirm or rule out SARS-CoV-2 infection [51]. For this reason, laboratory tests become necessary to identify a SARS-CoV-2 infection [52].

Given the low number of comorbidities reported by the participants, no significant differences emerged.

Cases and controls did not differ significantly by working macrocategories. The most represented workers were occupied in manufacturing activities, which were among the most affected sectors [53]. The ceramic sector, which is particularly developed in the province of Modena, was the most represented. Interestingly, there was a higher percentage of subjects employed in the ceramic sector among seropositives compared to seronegatives. In contrast, workers in the chemical–pharmaceutical and metal–mechanical sectors were less represented among seropositives. This may suggest that workers in the ceramic industry are at a higher risk compared to the other two occupational sectors, even if the reasons for this difference are not fully clear. Possible explanations can be found in the better attitude of chemical–pharmaceutical workers towards the wearing of protective masks, due to the consolidated habits aimed at protecting their airways from hazardous chemicals. Moreover, it is plausible that in this sector, more restrictive procedures have already been implemented to guarantee compliance with the quality parameters required for their production. An observation of a low COVID-19 risk has been recently reported for metal workers by Esposito et al., indicating a low spread of infections in this sector, probably thanks to the implementation of restrictive infection control measures that included the rigorous use of masks for all working hours [54].

The use of FFP2 masks and the frequency of hand hygiene were investigated as preventive strategies. The adoption of both measures seems to be protective against SARS-CoV-2 infection evaluated through serological testing, in line with other investigations [55,56,57]. Abboah-Offei et al. evaluated the recent literature on the efficacy of face masks against respiratory virus transmission, including coronaviruses. The authors demonstrated that regardless of the type, setting, or who wears them, face masks serve a dual preventive purpose: protecting oneself from getting a viral infection and protecting others [58]. They observed that the combined use of a face mask and hand hygiene further decreased the risk of transmission. Although several studies agree that the adoption of both preventive strategies is protective against SARS-CoV-2, the reverse is also true: improper mask use and suboptimal hand hygiene are risk factors [59].

In our study, folate concentrations were significantly lower in the cases’ serum than in controls. Deschasaux-Tanguy et al. reported that dietary intakes of folate were associated with a decreased probability of SARS-CoV-2 infection [60]. On the other hand, Akbari et al. found no significant difference in the folate serum levels between the control group and COVID-19 patients [61]. Nevertheless, our findings are in line with a recent study by Pandya et al., which suggests a protective role of folates against SARS-CoV-2 infection thanks to their high affinity bond with viral proteins. This bond with proteins such as furin, spike protein, RNA-dependent RNA polymerase (RdRP), and non-structural protein 3 (NSP3) might have an inhibitory effect on virus replication [27]. A similar outcome emerged from a computational study that showed that folic acid molecules interacted with the active site residues of furin [62]. Moreover, a study conducted on mice and cell lines found that folic acid treatment decreased the expression of ACE and reduced the spike protein binding ability [63]. Folate deficiency may also affect the immune system by inhibiting the capacity of CD8+ T lymphocyte cells to proliferate in response to mitogen activation [64]. Therefore, a diet rich in folates (green leafy vegetables, citrus fruits, and beans) could potentially have a protective effect against infection.

This study has some limitations, such as the inclusion of only one geographical area, the inability to assess when the infection occurred and how severe it was, the predominance of workers, and therefore age groups between 18 and 70 years. Only positivity or negativity to the serological testing was considered, while the study did not take into account the differences in the cases’ antibody titres. Although exposure to some occupational and environmental factors was evaluated, other factors that may affect the antibody response to SARS-CoV-2 (such as particulate matter or volatile organic compounds) were not considered. Also, the study was conducted without considering the virus variants. However, in the study period (October 2020–May 2021), in the Emilia-Romagna region, the infection was mainly associated with the wild-type virus and the Alpha (B.1.1.7) and Gamma (P.1) variants. In particular, wild-type SARS-CoV-2 circulated until April 2021, while both the Alpha and Gamma variants emerged in March 2021 and were still the most widespread at the end of the study period [65,66]. In addition, the limited sample size did not allow for stratified analysis, such as for some working categories (e.g., human health and social work activities). However, the sufficient number of other sectors has made it possible to highlight interesting information to evaluate the effect of preventive measures applied in the working environment to contain the spread of SARS-CoV-2 infection.

In conclusion, our case–control study suggests an association between folates, living place, smoking habit, preventive measures, and SARS-CoV-2 infection evaluated by antibody response. Adequate folate levels, living in rural areas, and good adherence to preventive strategies seem to be protective. Individuals who smoke at least one cigarette a day could be less susceptible to the SARS-CoV-2 infection evaluated by antibody response, but this claim is controversial and should be further investigated. Finally, workers in the ceramics sector seem to be at greater risk of SARS-CoV-2 infection, and they could consequently be subjected to specific preventive interventions by their companies.

## Figures and Tables

**Table 1:** Characteristics of the study participants. All percentages were calculated, excluding the missing data.

Characteristics	Cases (*n* = 166)	Controls (*n* = 239)
Sex *n* (%)		
Female	95 (57.2)	133 (55.7)
Male	71 (42.8)	106 (44.3)
Age (years) (M ± SD)	50.6 ± 12.4	50.6 ± 11.6
Age class *n* (%)		
<40	30 (18.1)	37 (15.6)
40–49	38 (22.9)	64 (27.0)
50–59	50 (30.1)	80 (33.8)
≥60	48 (28.9)	56 (23.6)
BMI *n* (%)		
Underweight (<18.5)	6 (3.6)	6 (2.5)
Normal weight (18.5–24.9)	95 (57.6)	125 (52.5)
Overweight (25.0–29.9)	44 (26.7)	84 (35.3)
Obesity (≥30.0)	20 (12.1)	23 (9.7)
Smoking habit *n* (%) *		
Smokers (≥1 cigarette/day)	19 (11.5)	48 (20.2)
Non smokers	146 (88.5)	190 (79.8)
Living place *n* (%) *		
Urban area	102 (61.8)	135 (57.0)
Rural area	40 (24.2)	65 (27.4)
Industrial area	6 (3.7)	1 (0.4)
Other	17 (10.3)	36 (15.2)
SARS-CoV-2 infection diagnosis confirmed with swab *n* (%) *		
Yes	116 (70.3)	30 (12.7)
No	49 (29.7)	206 (87.3)
Previous symptoms suspected for COVID-19 *n* (%) *		
Yes	140 (84.3)	120 (50.2)
No	26 (15.7)	119 (49.8)
Clinical conditions *n* (%)		
Diabetes	4 (2.4)	2 (0.8)
Respiratory disease	16 (9.7)	30 (12.6)
Cardiovascular disease	43 (26.1)	62 (26.1)
Cancer	17 (10.3)	26 (10.9)

* Statistically significant difference, *p* < 0.05 (smoking habit: *p* = 0.022; living place: *p* = 0.040; SARS-CoV-2 infection diagnosis confirmed with swab: *p* < 0.001; previous symptoms suspected for COVID-19: *p* < 0.001).

**Table 2:** Characteristics of study participants by occupational category using ISIC classification. All percentages were calculated, excluding the missing data.

Occupational Sector	Cases *n* (%)	Controls *n* (%)
Manufacturing activities (C)	71 (57.7)	96 (54.9)
Wholesale and retail trade; repair of motor vehicles and motorcycles (G)	9 (7.3)	16 (9.1)
Information and communication services; financial and insurance activities; real estate activities; professional scientific and technical activities; administrative and support service activities (J, K, L, M, N)	16 (13.0)	19 (10.9)
Public administration and defense; compulsory social security (O)	3 (2.4)	6 (3.4)
Education (P)	7 (5.7)	7 (4.0)
Human health and social work activities (Q)	6 (4.9)	10 (5.7)
Arts, entertainment, and recreation; other service activities (R, S)	3 (2.5)	6 (3.4)
Agriculture, forestry, and fishing; electricity, gas, steam, and air conditioning supply; construction; transportation and storage; accommodation and food service activities (A, D, F, H, I)	8 (6.5)	15 (8.6)

**Table 3:** Most represented sectors among manufacturing activities. All percentages were calculated, excluding the missing data.

Manufacturing Activities	Total *n* (%)	Cases (*n* = 71) *n* (%)	Controls (*n* = 96) *n* (%)
Ceramic	71 (42.5)	44 (62.0)	27 (28.1)
Chemical–pharmaceutical	39 (23.3)	5 (7.0)	34 (35.4)
Metal–mechanical	29 (17.4)	7 (9.9)	22 (22.9)
Other	28 (16.8)	15 (21.1)	13 (13.6)

**Table 4:** Characteristics of study participants by adherence to preventive measures in the workplace. All percentages were calculated, excluding the missing data.

Preventive Measures	Cases *n* (%)	Controls *n* (%)
FFP2 mask use		
No or rarely	89 (65.4)	115 (58.7)
Yes	47 (34.6)	81 (41.3)
Hand hygiene		
<5 times/day	54 (39.4)	61 (31.0)
≥5 times/day	83 (60.6)	136 (69.0)
Preventive measures overall		
Low adherence (no or rare use of the FFP2 mask and hand hygiene < 5 times/day)	36 (55.4)	42 (40.8)
High adherence (FFP2 mask use and hand hygiene ≥ 5 times/day)	29 (44.6)	61 (59.2)

**Table 5:** Concentration of additional biochemical parameters in serum stored after blood sampling for anti-SARS-CoV-2 antibody assays.

Biochemical Parameters	Cases Median (Q1; Q3)	Controls Median (Q1; Q3)
Total cholesterol (mg/dL)	194 (165; 234)	200 (164; 222)
HDL cholesterol (mg/dL)	56 (44; 69)	55 (43; 67)
LDL cholesterol (mg/dL)	109 (90; 138)	117 (95; 139)
Triglycerides (mg/dL)	98 (66; 142)	92 (68; 129)
Sideraemia (µg/dL)	97.0 (71.5; 124.0)	97.0 (74.0; 127.0)
Folates (ng/mL) *	2.6 (1.7; 4.2)	3.0 (2.1; 5.0)
Vitamin B12 (pg/mL)	369.1 (277.1; 464.0)	364.5 (277.0; 472.4)
Vitamin D (ng/mL)	22.1 (16.5; 27.9)	21.2 (14.7; 29.1)
Copper (µg/L)	1128.0 (970.0; 1261.0)	1121.0 (994.0; 1270.5)
Zinc (µg/L)	936 (828; 1040)	942 (836; 1064)
Nickel (µg/L)	0.73 (0.52; 0.95)	0.74 (0.56; 0.92)
Chromium (µg/L)	0.62 (0.52; 0.79)	0.61 (0.48; 0.74)

* Statistically significant difference, *p* < 0.05 (folates: *p* = 0.042).

## Data Availability

The data presented in this study are available on reasonable request from the corresponding author. The data are not publicly available due to privacy and legal restrictions.
